# Pulmonary embolism as a complication of an electrophysiological study: a case report

**DOI:** 10.1186/s13256-016-0872-0

**Published:** 2016-04-11

**Authors:** Sahela Nasrin, Fathima Aaysha Cader, Mohammad Salahuddin, Tahera Nazrin, Jabed Iqbal, Masuma Jannat Shafi

**Affiliations:** Department of Cardiology, Ibrahim Cardiac Hospital & Research Institute (ICHRI), Dhaka, Bangladesh

**Keywords:** Electrophysiological study, Deep vein thrombosis, Pulmonary Embolism

## Abstract

**Background:**

Electrophysiological studies have become an established practice in the evaluation and treatment of arrhythmias. Symptomatic pulmonary embolism as a result of deep vein thrombosis arising from multiple venous sheath femoral vein catheterization is an uncommon complication associated with it. We report the case of a 33-year-old woman who developed pulmonary embolism after an electrophysiological study, which was successfully treated at a cardiac hospital in Bangladesh.

**Case presentation:**

A 33-year-old Bangladeshi woman with hypertension and diabetes had initially presented with recurrent episodes of paroxysmal atrial fibrillation that manifested as palpitations for 2 years. Her atrial fibrillation was drug-refractory and could not be attributed to a treatable etiology. She had undergone an electrophysiological study at a different hospital, where right femoral venous catheterization was performed followed by the insertion of three venous sheaths. However, tachyarrhythmia could not be induced and a procedure to isolate the pulmonary vein was postponed because all the veins could not be isolated. Forty-eight hours later, she presented to our hospital with shortness of breath, chest heaviness, palpitations, and recurrent episodes of syncope.

She had normal coronary arteries and no other risk factors for venous thromboembolism. She was hemodynamically stable on examination. There was echocardiographic evidence of pulmonary hypertension and right ventricular dilatation and dysfunction. A computed tomography pulmonary angiogram confirmed pulmonary embolus in the descending branch of her left pulmonary artery, extending up to the segmental arteries.

Subsequently, a duplex ultrasound confirmed acute deep vein thrombosis affecting her right ilio-femoral segment. She was successfully managed with subcutaneous enoxaparin and oral warfarin (target international normalized ratio 2.5–3).

**Conclusions:**

Pulmonary embolism is a rare but serious complication that may occur in patients who undergo electrophysiological studies. Multiple venous sheaths inserted into the femoral vein and catheter-induced endothelial injury, further compounded by prolonged procedural time, may be responsible for the increased thrombogenicity leading to deep vein thrombosis and subsequent pulmonary embolism. An adequate observation time after the procedure and clinical alertness are necessary for rapid diagnosis and treatment.

## Background

Over the years, clinical electrophysiological studies (EPS) have become a recognized component of the diagnostic evaluation of and therapeutic strategy for patients with different cardiac arrhythmias [1–3]. Catheter ablation is a class IA recommendation for the treatment of paroxysmal and symptomatic atrial fibrillation (AF) that are refractory or intolerant to at least one class 1 or 3 antiarrhythmic medication. Pulmonary vein isolation (PVI) is the cornerstone of the strategy, although the procedure itself can be challenging: it requires considerable procedural time and operator expertise, and is limited by the infrequency with which AF initiation can be reproducibly triggered [4]. Depending on the type of procedure, age of the patient, and comorbidities, major complications occur in approximately 1.1 % of patients undergoing EPS, with a significantly higher complication rate of 3.1 % among those undergoing radiofrequency catheter ablation (RFCA) [5].

The procedure-related complications arising from clinical cardiac EPS include pulmonary embolism (PE), cardiac perforation, arterial injury, thrombophlebitis, systemic arterial embolism, and sometimes death [6]. EPS involve the percutaneous introduction of one or multiple catheters to record the electrical activity of the heart or to pace its different cavities. The introduction and manipulation of these catheters in arteries, veins, or cardiac cavities may activate the coagulation cascade, risking thromboembolism, such as deep vein thrombosis (DVT). Furthermore, withdrawal of catheters induces hemorrhage, usually limited by the compression of the site of venous or arterial puncture, leading to venous stasis and a further thrombogenic state [7].

Symptomatic PE following EPS is relatively uncommon, with a complication rate between 0 and 1.7 % [6, 8]. Despite its low incidence, it is a serious complication, with a mortality of greater than 15 % in the first 3 months following the diagnosis [9, 10]. Prompt diagnosis and management of venous thromboembolism (VTE) and subsequent PE can result in a good outcome [11]. A better understanding of the risk factors for PE and the proactive administration of thromboprophylaxis in high-risk patients may help minimize the risk of this complication [9]. We report the case of a woman who presented with DVT with PE, occurring as a complication of EPS. Our patient was successfully treated with subcutaneous enoxaparin and subsequent warfarin.

## Case presentation

A 33-year-old Bangladeshi woman with hypertension and diabetes had initially presented to a different hospital with intermittent episodes of palpitations for 2 years, with documented electrocardiographic (ECG) evidence of AF during the episodes. She had previously been on oral propafenone, amiodarone, and metoprolol, at separate times, but continued to experience paroxysmal AF.

She had a 2-year history of hypertension that was well controlled with metoprolol 50 mg twice daily and ramipril 5 mg once daily. She had type 2 diabetes mellitus, diagnosed 3 years previously, that was well controlled with metformin 500 mg twice daily. There was no evidence of diabetic nephropathy or microalbuminuria. Furthermore, other etiologies of AF, such as thyrotoxicosis, were excluded by her thyroid profile, which comprised normal levels of TSH, free triiodothyronine, and free thyroxine. Ischemic heart disease was excluded by a CT coronary angiogram, which revealed normal epicardial coronary arteries.

Given the indication of drug-refractory AF, our patient had undergone EPS at a different hospital from which she had been discharged 24 hours after the procedure. The EPS has comprised right femoral venous catheterization followed by the insertion of three venous sheaths into her femoral vein.

Because an inducible arrhythmia could not be reproduced, her pulmonary vein (PV) potentials were mapped and complete PV isolation was planned. But, because there was difficulty in isolating some of the PVs along with an extended procedural time, the procedure was abandoned. A plan was made to identify all PVs more specifically using other imaging techniques such as magnetic resonance imaging (MRI), prior to attempting the procedure again.

She presented to our hospital 48 hours after the EPS with shortness of breath and chest heaviness, associated with palpitations and recurrent episodes of syncope. When queried, she confirmed that these symptoms were not like those she had previously experienced during her paroxysms of AF, and reiterated that they only developed after the EPS.

She had no prior history of thromboembolic events, long flights, prolonged immobilization, or oral contraceptive use, and she denied any family history of thrombophilia. There was no evidence of either hypertensive or diabetic nephropathy. A urine dipstick test was negative for protein. A thrombophilia work-up revealed normal levels for protein C and S, antinuclear antibody, and anticardiolipin antibody, thus eliminating other causes of hypercoagulability as possible etiologies of thrombosis.

On examination, she was hemodynamically stable and heart and lung auscultation was unremarkable. There was no unilateral leg swelling. An ECG showed T inversions in leads III, aVF, and V1–4. It also revealed mild tricuspid regurgitation and a pulmonary artery systolic pressure (PASP) of 50 mmHg. Her right ventricle (RV) was dilated, with diameters measuring 38 mm at the basal level and 40 mm at mid-level, with a longitudinal measurement of 90 mm on the apical 4 chamber view. Tricuspid annular plane systolic excursion (TAPSE) was measured at 12 mm. Her left atrium (LA) was of normal size and measured 33 mm. Qualitative assessment revealed that right atrium was larger than her LA. There was no evidence of left ventricular (LV) hypertrophy. Her LV ejection fraction (EF) was 65 % and there were no regional wall motion abnormalities of her LV. There was no evidence of thrombus in any of the cardiac chambers.

**Fig. 1:**
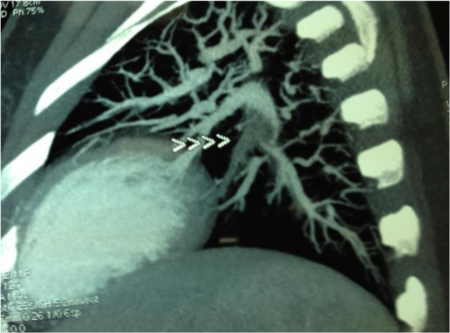
CT pulmonary angiography showing thrombus. CT pulmonary angiogram shows filling defects in descending branch of left pulmonary artery (arrow heads) extending up to segmental arteries, suggesting thrombus.

A CT coronary and pulmonary angiogram revealed normal coronaries with filling defects in the descending branch of her left pulmonary artery extending into the segmental arteries, suggesting thrombus (Fig. 1).

**Fig. 2:**
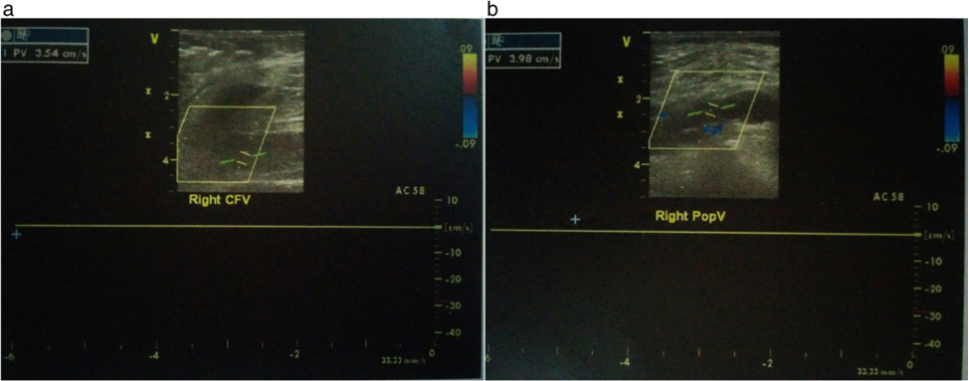
Duplex ultrasound scan of right lower limb showing deep vein thrombosis. Duplex scan shows absent blood flow in the right common femoral veins (panel **a**) and right popliteal vein (panel **b**), indicating deep vein thrombosis

Subsequently, a duplex ultrasound study of her lower limb venous system showed absent blood flow in her right external and common femoral veins, confirming acute DVT affecting her right ilio-femoral segment without signs of recanalization (Fig. 2).

Given the sequence of events and their temporal relationship with the EPS, and in the absence of other risk factors for DVT, it was established that the acute DVT occurred as a consequence of the femoral vein catheterization and multiple venous sheaths inserted for EPS, subsequently leading to PE. She was treated with subcutaneous enoxaparin 60 mg twice daily for 5 days along with the administration of oral warfarin. She continued taking the warfarin for 6 months, with a target therapeutic international normalized ratio of 2.5–3. She was asymptomatic at follow-up at six months, and, due to the resolution of the DVT, oral anticoagulation was discontinued.

## Discussion

Clinical cardiac EPS with or without RFCA is an important component of the diagnostic and therapeutic work-up of serious arrhythmias. RFCA following EPS has become the treatment of choice for a wide variety of arrhythmias, including AF, atrial flutter, and supraventricular and ventricular tachycardias [7].

Ablation strategies that target the PVs and/or PV antrum are the cornerstone for most AF ablation procedures [4]. However, unlike other supraventricular arrhythmias, the provocation of PV ectopy is a challenge. Direct catheter ablation can be limited by a paucity of spontaneous or inducible arrhythmias during the procedure and the infrequency with which AF initiation can be reproducibly triggered, as was the case in our patient [12]. Around 3 % of paroxysmal AF have no inducible ectopy-initiating AF [13]. This is further compounded by the difficulty of precise mapping within the venous structures [4].

The identification of arrhythmogenic PVs can be time-consuming, because focal activity can be difficult to observe or induce during the procedure [12]. Patients with paroxysmal AF usually have multiple PV foci in multiple veins, and many of these foci originate distally (that is, 2–4 cm) in those veins [12]. Thus, the goal of PV isolation is to produce complete electrical isolation of all PVs in the hope of abolishing the initiating triggers [13], because PVI of only arrhythmogenic veins has been shown to have limited success rate [12].

However, significant variability in PV anatomy and morphology exists, leading to technical difficulties. Difficulty in catheter navigation may represent inaccuracy in the electro-anatomic map or result from patient movement during the procedure. Cannulation of the right inferior PV in particular can be challenging, requiring a prolongation of total procedural time. Also, the more proximal PV ablation approach requires correct identification of the ostia and PV-LA junction to prevent pulmonary stenosis [12, 13]. Thus, recognition of the individual patient’s anatomy is critical to success. In our case, a further attempt at PVI was planned after pre-procedural imaging, and after we had the exact location of the pulmonary ostia.

PE complicated by EPS is rare, with previous studies demonstrating an overall incidence of 0–0.25 % [14–17]. PE following EPS can be either benign and asymptomatic, or symptomatic with life-threatening consequences requiring urgent intervention [8, 11, 14, 15]. There are various risk factors associated with VTE, including obesity, immobilization, hospitalization, malignancy, long flights, the use of the combined oral contraceptive pill, and central venous catheterization [18]. Having ruled out the other risk factors associated with VTE in our patient, her recent femoral vein catheterization for EPS remained as the evident cause for the development of DVT and PE. Furthermore, the prolonged procedural time with *in situ* venous sheaths could have been an additional factor contributing to the development of venous thrombosis.

PE after EPS is frequently underestimated [19], with some authors even advocating same day discharge after EPS [16]. Primm *et al*. [19] reported that 12 % of patients had new perfusion defects detected by ventilation-perfusion lung scans 1 day after routine right-heart catheterization, suggesting that PE may be more common than previously appreciated.

The source for PE is often attributed to a DVT arising from the punctured femoral vein [14]. Venous catheterization and the use of multiple venous sheaths inserted into a single femoral vein is an essential component of most EPS and RFCA [9, 14]. Multiple intracardiac catheters are often necessary for EPS and RFCA therapy, and this almost always requires multiple venous sheath placements in a single femoral vein [20].

Chen and colleagues [20] reported that such use of multiple (that is, up to three) femoral sheaths in a single femoral vein during EPS resulted in a high incidence (19.2 %) of non-occlusive DVT detected by duplex ultrasonography on the day following the EPS. Although the outcome of non-occlusive DVT in the study by Chen *et al*. [20] was largely benign in most patients, and they concluded that short-term placement of multiple venous sheaths in a single femoral vein appeared to be safe, they advised close follow-up to avoid potential vascular complications.

Our patient had three sheaths inserted into her right femoral vein. She had a relatively long procedure time of approximately 70 minutes. Following the EPS, she stated that she had a relatively long duration of hemostasis (approximately 3.5 hours) with sandbag application. This may have compressed her femoral vein, leading to venous stasis. Authors have suggested that both venous stasis and injury to vascular endothelium caused by multiple venous sheath placements may be possible mechanisms for the formation of DVT and subsequent PE [7, 14].

The mechanisms of thromboembolism during EPS and peri-ablation have been open to much speculation [21]. Although the pathophysiology of thromboembolism may be multifactorial, it is the fulfillment of Virchow’s triad in the context of an electrophysiological procedure that leads to a prothrombotic or hypercoagulable state resulting in VTE [7, 21].

Davutoglu *et al*. [22] reported a significant increase in occult femoral vein thrombosis following the use of multiple femoral venous sheaths, without using heparin, in patients undergoing EPS. The incidence of femoral vein thrombosis was high (62.5 %); however, no patients showed clinical features of PE. Furthermore, a prophylactic fixed dose of body weight-independent low-molecular-weight heparin (LMWH) significantly decreased the risk of femoral thrombosis in these patients (18 %) [22]. They attributed the main risk factors for DVT in EPS to be catheter thrombogenicity, lack of anticoagulation, and occlusion of the lumen secondary to multiple introducer sheaths. In cases of ablation, the increased activation of the clotting system could be an additional factor for thrombosis [22]. In fact, Chen *et al*. [5] reported that RFCA presents a significantly higher overall complication rate in comparison to EPS alone (3.1 % versus 1.1 %, respectively, *P* = 0.00002).

Typically, a PE can develop from 8.5 hours to 14 days after a EPS procedure [9, 15], thus warranting a minimum period of observation for complications after procedure. However, Marijon and colleagues [16] suggested that same-day home discharge after 4–6 hours of observation is safe for uncomplicated RFCA, despite the fact they noted symptomatic delayed PE.

Our patient presented with PE 48 hours after EPS, and 24 hours after discharge. Thus, we believe that a longer observation period may be needed in some patients, particularly those who have a longer duration of hemostasis. Additionally, a duplex ultrasonography of the lower limbs prior to discharge may be prudent to evaluate the formation of thrombus at the site of the punctured femoral veins, especially in those patients being discharged on the same day.

There is also the alternative issue of the role of anticoagulation during and following electrophysiological procedures in preventing VTE after EPS. There is currently no definitive consensus regarding the use of anticoagulants either during or after routine EPS, including those involving catheter ablation [7]. A policy statement by the North American Society of Pacing and Electrophysiology (NASPE) suggested the application of heparinization for catheter manipulation during left-sided RFCA [1].

Evidence from this case and others [11, 14] suggests that, although rare, VTE and PE are potentially life-threatening in certain patients after EPS. Based on the frequency and potential long-term complications of PE, there is a pressing need for more evidence-based consensus concerning the issues of anticoagulation and the prevention of VTE during routine EP procedures [11].

## Conclusions

Although rare, PE arising from DVT may occur even in patients undergoing EPS with a short procedure time. A prolonged procedure time may further increase the risk of developing DVT. Furthermore, the symptoms of PE may be easily misinterpreted to be those of the initial arrhythmia; as such, prompt diagnosis and treatment requires a high index of suspicion for PE in the case of new symptoms in post-EPS cases. There is also a close relationship between EPS and thrombus formation and, thus, there is a need to balance the risks between thromboembolism and bleeding. Endothelial injury resulting from multiple venous sheaths and prolonged hemostatic mechanisms may be responsible for the formation of DVT. There are no guidelines on the use of antithrombotic therapies in the setting (that is, before, during, and after) of EPS, and an evidence-based consensus is warranted.

## Consent

Written informed consent was obtained from the patient for publication of this case report and any accompanying images. A copy of the written consent is available for review by the Editor-in-Chief of this journal.

## Abbreviations

EPS: Electrophysiological study
AF: atrial fibrillation
PVI: pulmonary vein isolation
RFCA: radio frequency catheter ablation
DVT: deep vein thrombosis
PE: pulmonary embolism
ECG: electrocardiography
PASP: pulmonary artery systolic pressure
RV: right ventricle
TAPSE: tricuspid annular plane systolic excursion
LA: left atrium
LV: left ventricle/ventricular
EF: ejection Fraction
PV: pulmonary veins
VTE: venous thromboembolism
LMWH: low molecular weight heparin
NASPE: North American Society of Pacing and Electrophysiology
